# Chronic bronchitis in oil industry workers exposed to hydrocarbons: role of cigarette smoking and gender differences

**DOI:** 10.1186/1617-9625-12-S1-A32

**Published:** 2014-06-06

**Authors:** Eniko Viragh, Karoly Viragh, Horia Suciu

**Affiliations:** 1 University of Medicine and Pharmacy, Targu Mures, 540139, Romania; 2 Olive View UCLA Medical Center, Sylmar, Los Angeles, California, 91342, USA; 3 Factory Medical Office, Suplacu de Barcau, 417536, Romania

## Background

The purpose of the study was (1) to determine the prevalence of smoking and chronic bronchitis in oil industry workers exposed to hydrocarbons, and (2) to evaluate the effect of cigarette smoking and gender differences in the development of chronic bronchitis.

## Materials and methods

A retrospective case-control study was performed. Industry workers from an oil refinery were monitored for a 6yrs period. The air levels of aliphatic and aromatic hydrocarbons were monitored. During this period, all workers received regular clinical examinations, which included evaluation for smoking status, occupational exposure to hydrocarbons (aliphatic and aromatic), and the presence and severity of chronic bronchitis. The workers were divided into two groups of approximately 100 patients each: Group (1) males exposed to hydrocarbons; and Group (2) females exposed to hydrocarbons. Appropriately-matched control groups were also selected from non-exposed workers, who matched in age, gender, work history and lifestyle. The prevalence of smoking and chronic bronchitis was determined in each group. Linear regression analysis was used to assess the correlation between exposure to hydrocarbons and effects (chronic bronchitis), as well as smoking, and severity of chronic bronchitis.

## Results

During the studied period, hydrocarbon levels in the air of all workplaces were persistently high: aliphatic hydrocarbon levels were 1800mg/m3 (maximum allowable concentration, MAC = 1000mg/m3) and aromatic hydrocarbon levels were 341.5ng/m3 (MAC=150ng/m3, marker: 3,4-benzo[a]pyrene). The prevalence of chronic bronchitis in hydrocarbon-exposed females was 16.2%, higher than in exposed males 11.3%. Smokers in each group had significantly higher rates of chronic bronchitis than non-smokers. Linear regression analysis showed strong correlation between exposure and chronic bronchitis: males (r=0.61) and females (r=0.72).

## Conclusions

There is high prevalence of smoking and respiratory disease in exposed oil industry workers. Cigarette smoking is an important risk factor for respiratory disease and acts in combination with hydrocarbons in exposed industry workers. Females had more severe disease than males. Cigarette smoking may act as a confounder in the assessment of the severity of occupational disease related to hydrocarbon exposure in industry workers. The goal for all facilities and workers is to minimize smoking and occupational exposure to noxious agents.

